# Pre-vaccine circulating group a rotavirus strains in under 5 years children with acute diarrhea during 1999–2013 in Cameroon

**DOI:** 10.15761/VRR.1000120

**Published:** 2017-07-25

**Authors:** Paul Koki Ndombo, Valantine N. Ndze, Charles Fokunang, Taku Nadesh Ashukem, Angeline Boula, Mina N. Kinkela, Corlins E. Ndode, Mapaseka L Seheri, Michael D. Bowen, Diane Waku-Kouomou, Mathew D. Esona

**Affiliations:** 1 Faculty of Medicine and Biomedical Sciences, University of Yaoundé I, Yaounde, Cameroon, South Africa; 2 Rotavirus National Reference Laboratory, Mother and Child Centre of the Chantal Biya Foundation, Yaoundé, Cameroon, South Africa; 3 South Africa Medical Research Council/Diarrhoeal Pathogen Research Unit, Department of Virology, Faculty of health Sciences, Sefako Makgatho Health Sciences University, Medunsa, Pretoria, South Africa; 4 Centers for Disease Control and Prevention, Atlanta, GA, USA

**Keywords:** rotavirus, genotypes, Cameroon, vaccine

## Abstract

The aim of this review was to assess all the studies on rotavirus G and P characterization during the pre-vaccine period (1999–2013) in Cameroon to have a better basis for post-vaccine introduction evaluations.

A retrospective study was done through a comprehensive review of published (PubMed, Google Scholar) and accessible unpublished data on rotavirus G and P genotypes circulating in five regions of Cameroon. Descriptive data were expressed as frequencies tables and proportions.

A total of 1844 rotavirus positive cases were analyzed. In all, 1534 strains were characterized for the P (VP4) specificity. Six different VP4 genotypes were observed, including P [4], P [6], P [8], P [9], P [10] and P [14]. The most predominant P genotypes were P [8] at 42.6%, and P [6] at 37.9%. Mixed infections were observed at 5.3%, whereas 4.1% of the strains were P non-typeable. A total of 1518 rotavirus strains were characterized for the G (VP7) specificity. VP7 genotypes G1, G2, G3, G4, G5, G6, G8, G9, G10 and G12 were observed. G1 (35.3%), G3 (19.5%), G2 (14.9%) and G12 (10.1%) were the predominant G genotypes while G5 and G10 were least prevalent at 0.06% each. Approximately 5.1% of all strains were G non-typeable whereas 5.3% were mixed G genotypes. A total of 1472 strains were characterized for both G and P genes, from which 38 different G–P combinations were observed. Overall, G1P [8] (22%) was identified as the predominant rotavirus strain circulating in Cameroon followed by G3P [6] (15%).

In conclusion, we observed that the genotypes identified in Cameroon during 1999–2013 were partially covered by the two WHO recommended rotavirus vaccines. This review provides comprehensive up-to-date information on rotavirus strain surveillance in Cameroon during the pre-vaccination era.

## Introduction

Group A Rotavirus (RV) is the leading cause of severe acute gastroenteritis in infants and young children worldwide but the case fatality rate among hospitalized children is higher in the poor, developing countries of Africa and Asia [1]. RV gastroenteritis (RVGE) can lead to severe dehydration [2] and consequently, it is estimated that RVGE accounts for about 39% of all deaths due to diarrhea in children <5 years of age worldwide [3]. Globally, RV infections result in an estimated 23 million outpatient visits and 2.3 million hospitalizations each year [4]. Global estimates show that the number of rotavirus deaths in children <5 years of age declined from 528 000 (range, 465 000–591 000) in 2000 to 215 000 (range, 197 000–233 000) in 2013 [5]. The majority (56%) of rotavirus deaths occurred in countries of sub-Saharan Africa, a region that accounted for all 10 countries in 2013 with rotavirus mortality rates >100 per 100 000 [5]. Nigeria alone accounts for 14% (30800) of global RV deaths and has the highest RV related mortality rate in Africa with >100 deaths per 100,000 children < 5 years of age, while Cameroon has a RV related mortality rate in the range of 50–100 deaths per 100 000 children < 5 years old [5].

RV are members of the family *Reoviridae* and contains 11 segments of double-stranded RNA (dsRNA) genome encoding six structural viral proteins (VP1-VP4, VP6 and VP7) and five or six non-structural proteins (NSP1-NSP5/6). Each RV gene segment is monocistronic with the exception of gene segment 11 which in some strains has an additional overlapping open reading frame (ORF) which encodes a tentative dispensable protein, NSP6 [6]. Six RV G-P constellations (G1P [8], G2P [4], G3P [8], G4P [8], G9P [8], and G12P [8]) are common globally, and are the prime targets for vaccine development [7]. Epidemiological studies around the world have demonstrated that RV strains bearing these 5 constellations are responsible for most infections. However, other genotypes, such as G5, G6, G8, G10, G12, P [9], P [11], P [14] and P [19], have also been detected in different areas of the world [8]. Studies from Africa reported high prevalence of genotypes G8 and P [6] in various combinations suggesting that both of these genotypes should be considered common in Africa. These studies concluded that the predominant strains circulating across Africa during 1996–1999 were G1P [6] and G3P [6] strains [9]. In Cameroon, Esona et al., 2010 showed that the predominant G-P combination detected in Western Cameroon during 1999–2000 rotavirus seasons was G1P [8] [10]. However, in Yaoundé (the Central region of Cameroon) during 2008–2010, genotype G9P [8] was predominant [11]. Ndze and coworkers showed that genotype G12 was predominant in the Far North and NorthWest regions of Cameroon during 2010–2011 [12].

Two new live-attenuated oral vaccines from Merck (RotaTeq^®^) and GlaxoSmithKline (Rotarix^®^) have been licensed in more than 100 countries and have been introduced into routine immunization programs in the United States and other countries in Latin America, Europe, Africa and Asia. Rotarix^®^ is a monovalent human G1P [8] vaccine and RotaTeq^®^ is a pentavalent vaccine consisting of 5 human-bovine reassortant strains expressing G1, G2, G3, G4 and P [8]. Post marketing surveillance of vaccine impact has demonstrated the great public health benefit of RV immunization in developed and developing countries [13]. These outcomes are consistent with the results of clinical trials, which indicated an efficacy for the Rotarix^®^ and RotaTeq^®^ vaccines against severe gastroenteritis of at least 85% in high income countries [14]. In contrast, clinical trials have indicated that the vaccines are much less efficacious in some low-income countries, for reasons that are not fully understood [15].

In April 2014, Cameroon introduced the Rotarix^®^ vaccine into its expanded program on immunization. Recent studies in Cameroon showed the emergence of uncommon rotavirus genotypes such as G6P [6], G9P [6] [11]. These genotypes are not present in the formulation of the two available RV vaccines. As the emergence of new genotypes may cause problems with vaccine effectiveness, it was therefore imperative to carry out a review of all the studies carried in Cameroon on rotavirus G and P characterization during the RV pre-vaccine period (1999–2013). These data will help monitor the impact of vaccination and RV genotype evolution post vaccine introduction.

## Methods

A retrospective study was done through an exhaustive review of published (PubMed, Google) and accessible unpublished data on RV genotypes in children < 5 years circulating in Cameroon from 1999 to 2013. Data were extracted from 4 published articles reporting rotavirus data from five regions in Cameroon: (West and South West regions) [10], (Far North and North-West regions) [12], (Centre region) [11]. Unpublished data for 2013 were also extracted from the registers of the rotavirus sentinel site at the Mother and Child Centre of the Chantal Biya Foundation in Yaoundé. Excluded from this work were studies on children > 5 years old and adults.

### Recruitment of patients

The procedures for obtaining informed consent from parents or guardians and patient recruitment have been described elsewhere [10–12]. Stool samples were collected from children <5 years of age who presented with acute diarrhea at the Regional Hospital Maroua and the Domayo Djama integrated health center in the Far North region, at the Regional Hospital Bamenda and the Esu integrated health centre in the North West region, at the Presbyterian Hospital Kumba, Southwest region, and at the Cabinet des Soins du Secour clinic, Bafoussam, West region [10, 12]. In the Centre region, inclusion and exclusion criteria for diarrhea cases were applied as specified in the AFRO standard operating procedures, which were based on the WHO Generic Protocol [16]. Children < 5 years of age who were hospitalized at the Mother and Child Centre of the Chantal Biya Foundation, Gyneco-obstetric and Pediatric Hospital Yaounde, and at the Biyem-assi District Hospital with the primary diagnosis of acute gastroenteritis also were recruited for the study [11].

Stool samples were either stored at −20°C or −70°C and transported on ice to: i) the MRC/MEDUNSA Diarrhoeal Pathogens Research Unit, Pretoria, South Africa, where they were processed for genotyping using reverse transcription-polymerase chain reaction (RT-PCR) and sequencing; ii) the Virology unit of Mother and Child Centre of the Chantal Biya Foundation for RV detection and RT-PCR genotyping, or iii) the Institute of Veterinary Medical Research, Budapest, Hungary for RT-PCR and sequence analysis.

### Rotavirus screening and Genotyping

Diagnosis of RV infection was determined using an antigen capture enzyme immunoassay IDEIA/PROSPECT [11] or DAKO diagnostics kit [10,11] or by VP6 RT-PCR [12].

From the rotavirus positive samples viral RNA was extracted from 140 μl of 10% stool suspension using the QIAamp viral RNA Mini kit (Qiagen, Inc., Valencia, CA, USA) according to manufacturer ’s instructions and stored at −80°C.

The extracted dsRNA of each strain was denatured at 97°C for 5 min and then RT-PCR was carried out using a One-Step RT-PCR kit according to manufacturer’s instructions. Previously published consensus primers [15–17] were used for the amplification of the VP4 and VP7 gene segments. Genotyping of the VP4 and VP7 genes was performed by semi-nested RT-PCR assay [15,16,18] using first round RT-PCR products generated by consensus primers. For G and P-type determination, a series of multiplexed type-specific primers were utilized (including G1–G4, G8, G9, G10 and G12 and P [4], P[6], P[8], P[9], P[10] and P[11] [15,17,18]. For a randomly selected subset of strains representing distinct genotypes, sequencing was carried out to confirm G and P typing data obtained by the multiplex genotyping PCR assay. Descriptive data were expressed as frequency tables and proportions.

## Current status of knowledge

**Figure 1:**
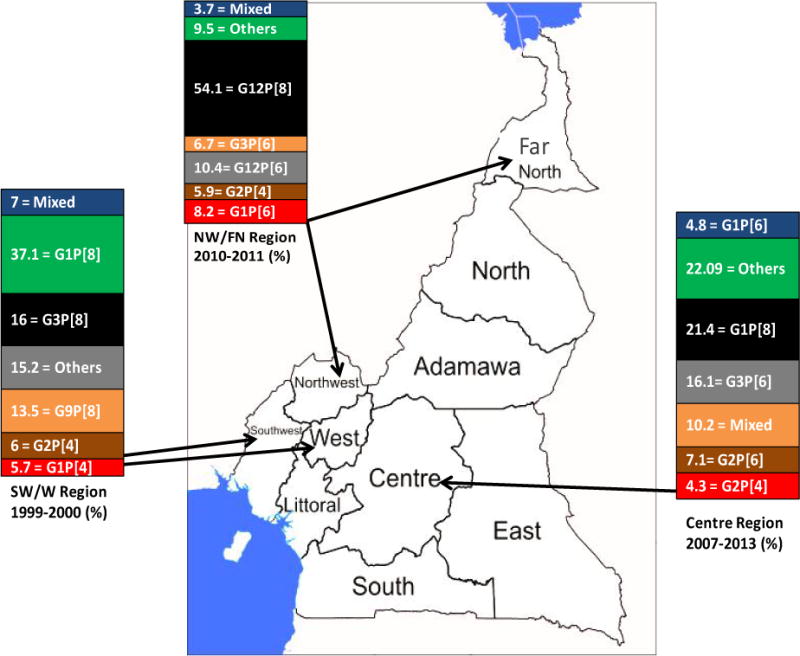
Percentage prevalence of rotavirus strains with respect to regions and periods of study. Only the five most prevalent strains per region are shown for each region and others represent the less common strains. SW = South West, W = West, NW = North West, FN = Far North.

A review of the literature and unpublished data showed that 4843 cases of gastroenteritis among children < 5 years were investigated for RV from June 1999 to December 2013 in the five regions of Cameroon (Figure 1). One thousand-eight hundred forty-four were positive for RV (Table 1) and 1585 were subjected to RV VP7 (G) genotyping and while 1537 were subjected to VP4 (P) typing by either a semi-nested RT-PCR or by sequencing.

Table 1 illustrates the yearly RV prevalence between 1999 and 2013 by age, with a standing RV prevalence in Cameroon of 38.1% (1844/4843). The highest RV prevalence in Cameroon was observed in 2011 (45.3%) and the lowest in 2009 (21.2%). The most affected age group was 6–11 months, except in 1999–2000, where the most affected age group was 12–23 months.

### Prevalence of individual P Types

A total of 1534 strains were characterized for P specificity. Seven different VP4 genotypes were observed, including P [4], P [6], P [7], P [8], P [9] P [10] and P [14]. The predominant P genotypes were P [8] at 42.6%, followed by P [6] (37.9%), P [4] (9.8%), P [9] (0.1%) then P [10] and P [14] at 0.06% each (Table 2). Mixed infections were observed at 5.3%, whereas 4.1% of the strains were non-typeable.

### Prevalence of individual G Types

A total of 1518 RV strains were characterized for the G specificity. In all, ten different RV VP7 genotypes were observed, including G1, G2, G3, G4, G5, G6, G8, G9, G10 and G12. G1 was the most predominant G genotype (35.3%) followed by G3 (19.5%) and G2 (14.9%). G12 at 10.1%, G9 and G4 at 3.4%, G8 at 2.3%, G6 at 0.8% while G5 and G10 were least at 0.06% each. Five point one percent of all strains were non-typeable whereas 5.3% presented mixed G genotypes (Table 2).

### G-P combinations

A total of 1472 strains were successfully characterized for both the G and P genes. Thirty-eight different G-P combinations were observed. G1P [8] (22%), G3P [6] (15%), G12P [8] (8%), and G2P[6] (7%) were predominant (Table II) whereas G1P[10], G3P[4], G4P[4], G5P[8], G6P[8], G8P[4], G8P[8], G9P[4], G9P[14], G10P[8] and G12P[4] exhibited prevalence’s below 0.5% and which represent the uncommon strains in this region.

Figure 1 illustrates the diversity of the five most common RV strains in six regions in Cameroon. In the South West and West regions, G1P [4], G1P [8], G2P [4], G3P [8] and G9P [8] were the five most common strains with G1P[8] (37.1%) being predominant, while in the Centre regions G1P[6], G1P [8], G2P [4], G2P [6] and G3P[6] were the most common strains with G1P [8] (21.4%) also being the predominant strain during the study period. In the North West and Far North regions, genotypes G1P [6], G2P [6] G3P [6], G12P [6], G12P [8] were the most common strains with G12P [8] (54.1%) being the predominant rotavirus strain in these regions.

## Discussion

The aim of this study was to review the RV genotypes circulating in Cameroon before the introduction of the monovalent Rotarix^®^ vaccine in the country. Despite the limited number of studies on the area in Cameroon, some important findings relevant to RV diversity were made in this present review.

In contrast to Europe [19], in Cameroon, the predominant G genotypes, G1, G2, G3, G4 and G9, dropped from 88.0% in 1999 to 73.2% in 2013. This lower prevalence might be due to the emergence of other genotypes such as G12 recently identified in northern Cameroon, which accounted for 10.1% of circulating G genotypes.

P[6] accounted for one third of P genotypes as reported in other studies in Africa [20]. This calls for more attention as to its status in the diversity of RV strains since P [6] is not included in the vaccines currently in use and therefore may have an impact on vaccine effectiveness in Cameroon. Notwithstanding, we are expecting that heterotypic immunity may provide partial protection against P [6] strains and other genotypes not included in the vaccines.

The high diversity of RV strains in Cameroon results in lower prevalence of certain strains. For instance, the six globally common rotavirus G-P combinations, G1P [8], G2P [4], G3P [8], G4P [8], G9P [8] and G12P[8], were responsible for 41.2% of the RV diarrhea among children less than 5 years in Cameroon. Meanwhile, Norma and Yasutaka reported a prevalence of over 50% for these RV strains in Africa [20]. It should be noted that the incidence of G6, G12 and P [6] strains has increased considerably in the past 5 years, which could change the distribution of rotavirus G-P constellations in Cameroon. The differences in the predominant strains in the different regions shows that predominant strains might differ in the regions of Cameroon.

Unusual RV strains such G1P [4], G1P [10], G2P [8], G3P [4], G4P [4], G5P [8], G6P [8], G8P [4], G9P [14], G10P[8] and G12P[4] represented about 7.0% of all the strains, probably due to reassortment resulting from mixed rotavirus infections [21]. The impact of unusual strains on RV evolution has yet to be determined, however, such strains could provide the opportunity for the introduction of novel P or G genes into human population via reassortment events [22]. The non-typeable strains included in the study were the RV strains which could not be typed either for any of the G (5.1%) or P (4.1%) genotypes or for both genotypes (1.3%) which is lower than that observed (6.1% G-P non-typeable) in neighboring Nigeria [23].

Overall RV genotype G1P [8] was the predominant strain circulating in Cameroon as observed in most studies in Africa [13], Asia [24], the United States [25], and Europe [26]. This strain is the sole genotype of the Rotarix^®^ vaccine [27]. The next most frequently observed strain was G3P [6] (15.0%) instead of G2P [4], which is considered the second most important strain worldwide after G1P [8] [40]. G3P [6] was also reported in Ethiopia from 2008 to 2009 [28] and Ghana in 2009 [29] as the most predominant RV strain. The higher proportion of G3P [6] can be due to the increase in the incidence of the P [6] strains in the past 4 years in Cameroon and which could also have been influenced by the close inter-relationship of human and animals as observed in the African setting [30]. Another issue contributing to the apparent increase detection of P [6] could also be as a result of improved genotyping techniques and number of samples being genotyped.

Emerging genotypes such as G6, G8 and G12 in combination with P [4], P [6] and P [8] were observed in Cameroon during this study period. Also, a P [14] genotype was identified in combination with G9. P[14] is commonly found in animals [31]. The emergence of these new strains might represent dynamic molecular events occurring in Cameroon which generated RV reassortants. It will be important to monitor the spatiotemporal dynamics of emerging strains in Cameroon, which might become predominant after vaccine introduction. The detection of emerging strains re-enforces the need for enhanced RV surveillance in humans and animals as unusual RV strains commonly found in animals are increasingly being detected in humans [32]. Complete genome studies are needed to understand better the full picture of the circulating RV strains in Cameroon.

## Conclusion

This study shows a great diversity of RV strains circulating in Cameroon among which G1P [8] and G3P [6] are predominant. The strains circulating in Cameroon shared at least one genotype with RV vaccines recommended for routine immunization in all countries by the WHO. However, the emergence of new genotypes such as G6P [6] and G9P [14] could be problematic in terms of theoretical vaccine efficacy [33]. For this reason, it is critical to continue the surveillance of RV strains in Cameroon. This review provides comprehensive, up-to-date data on circulating RV strains in the human population in Cameroon before vaccine introduction.

## Figures and Tables

**Table 1:** Yearly distribution of rotavirus positive cases by age group, Cameroon 1999–2013.

Age (Months)	Rotavirus positive by year (%)								
	1999/2000	2007	2008	2009	2010	2011	2012	2013	Total
**0–5**	1 (0.5)	14 (28.0)	32 (27.1)	11 (31.4)	63 (24.5)	113 (32.2)	119 (33.4)	153 (31.7)	506 (27.4)
**6–11**	34 (17.4)	14 (34.0)	54 (45.8)	14 (40.0)	112 (43.6)	149 (42.5)	166 (46.6)	237(49.2)	783 (42.5)
**12–23**	74 (37.9)	17 (30.0)	28 (23.7)	10 (28.6)	63 (24.5)	61 (17.1)	61 (17.1)	78 (16.2)	390 (21.1)
**23–35**	33 (16.9)	15 (4.0)	3 (2.5)	0 (0.0)	13 (5.0)	7 (2.0)	7 (2.0)	12 (2.5)	89 (4.8)
**36–47**	29 (14.9)	2 (4.0)	1 (0.9)	0 (0.0)	3 (1.2)	3 (0.8)	3 (0.8)	1 (0.2)	45 (2.4)
**48–59**	24 (12.3)	0 (0.0)	0 (0.0)	0 (0.0)	3 (1.2)	0 (0.0)	0 (0.0)	1 (0.2)	31 (1.7)
**Total**	**195 (21.9)**	**50 (44.5)**	**118 (42.8)**	**35 21.2)**	**257 (43.0)**	**351 (39.1)**	**356 (45.3)**	**482 (43.1)**	**184 (38.1)**

**Table 2:** Distribution of group A Rotavirus G-P genotypes in Cameroon from1999–2013.

VP4 P-types (%)	VP7 G-types (%)											
	G1	G2	G3	G4	G5	G6	G8	G10	G12	GMIX	GNT	Total
**P [4]**	62 (4)	73 (5)	4 (0)	1 (0)	0	0	7 (0)	0	4 (0)	2 (0)	2 (0)	157 (9.8)
**P [6]**	85 (5)	109 (7)	243 (15)	40 (3)	0	10 (1)	17 (1)	0	28 (2)	43 (3)	23 (1)	607 (37.9)
**P [8]**	358 (22)	26 (2)	57 (4)	12 (1)	1 (0)	2 (0)	5 (0)	1 (0)	121 (8)	29 (2)	32 (2)	682 (42.6)
**P [9]**	0	0	0	0	0	0	0	0	0	0	2 (0)	2 (0.1)
**P [10]**	1 (0)	0	0	0	0	0	0	0	0	0	0	1 (0.06)
**P [14]**	0	0	0	0	0	0	0	0	0	0	0	1 (0.06)
**P [MIX]**	38 (2)	22 (1)	2 (0)	1 (0)	0	0	8 (0)	0	2 (0)	6 (0)	6 (0)	84 (5.3)
**P [NT]**	20 (1)	8 (0)	6 (0)	1 (0)	0	0	0	0	4 (0)	5 (0)	5 (0)	66 (4.1)
**Total**	**564**	**238**	**312**	**55 (3.4)**	**1 (0.06)**	**12 (0.8)**	**37. (2.3)**	**1 (0.06)**	**159 (9.9)**	**85 (5.3)**	**82 (5.3)**	**1600 (100)**

## What is already known on this topic

- Group A rotavirus causes acute gastroenteritis and most of deaths occur in children below five years in developing countries. In Cameroon, during the pre-vaccine period, rotavirus prevalence stood at 38.1%.

## What this study adds

- This review suggest that increases in the incidence of certain rotavirus strains such as VP7 G6, G12 and VP4 P [6] and the emergence of P [14] strains might represent dynamic molecular events occurring in this region leading to a great diversity of rotavirus strains in Cameroon.
